# Development of biodegradable Mg-Zn-Zr-based temporary implant materials through multidirectional forging and laser shock peening

**DOI:** 10.1038/s41598-025-25578-0

**Published:** 2025-11-24

**Authors:** S. Aditya Kudva, Gajanan Anne, Prakash Kumar, Ashwini Prabhu, S. Ramesh

**Affiliations:** 1 Department of Mechanical Engineering, Research Center, Shri Madhwa Vadiraja Institute of Technology and Management, Bantakal, Udupi, Visvesvaraya Technological University (VTU), Belagavi, 590018 Karnataka India; 2 Department of Mechanical and Industrial Engineering, Manipal Institute of Technology, Manipal Academy of Higher Education, Manipal, 576104 Karnataka India; 3 UnivLabs Technologies Private Limited, Gurugram, 122003 Haryana India; 4 Yenepoya Research Centre, Yenepoya (Deemed to be University), Deralakatte, Mangalore, 575018 Karnataka India; 5 School of Computer Science and Engineering, RV University, Bangaluru, 560059 India

**Keywords:** Biodegradable mg based alloy, Implant materials, Multidirectional forging, Laser shock peening, Cytotoxicity, Engineering, Materials science

## Abstract

Biodegradable magnesium alloys have been a fascinating but challenging research arena, as development of such alloys for clinical applications can be a game changer in orthopedics, while controlling the degradation rate of magnesium alloys in physiological conditions is an area of concern. In quest of solution to the existing issue, this study involves development of biodegradable Mg-4Zn-0.6Zr alloy subjected to multidirectional forging (MDF) for bulk property improvement and laser shock peening for surface property improvement. The MDF process was carried out for 5 passes and resulted in significant grain refinement of the alloy from 180 ± 12 μm to 16 ± 5 μm after 5 MDF passes as observed through Scanning Electron Microscope (SEM), Laser Shock Peening (LSP) induced further grain refinement in nano scale as evident through Transmission Electron Microscope (TEM). After 5 MDF passes, the average microhardness of the alloy increased to 101.8 HV due to grain refinement and strain, further rising to 112.6 HV post-LSP from additional grain refinement and dislocations, while tensile tests showed a 141% improvement in Ultimate Tensile Strength (UTS) after 3 MDF passes compared to the homogenized state. The results of the electrochemical corrosion tests indicated that both MDF and LSP resulted in the least corrosion rate, about a factor of one order of magnitude reduction in comparison to the homogenized sample. The key results including mechanical properties, corrosion behaviour in Hanks Balanced Salt Solution and cytotoxicity evaluation employing MG-63 osteoblast-like cells suggest that the considered Mg-4Zn-0.6Zr alloy could be a worthy candidate to be further explored as a bioimplant material.

## Introduction

 Magnesium (Mg), the lightest commercial structural material^1,2^, is gaining interest as its properties like high specific strength, damping capacity, castability, and machinability^3–5^. Its biodegradable properties have garnered significant value in the biomedical sector, particularly for bone fixation devices like bone plates, screws, pins, and cardiovascular stents^6,7^. The global market for orthopaedic implants is growing, offering high-quality, functional materials that improve physical and emotional health^8^. The optimal biodegradable implant encourages osteointegration, boosts bone healing, and provides mechanical support during the early implantation phase. Mg implants are biodegradable, meaning no additional surgery is needed after healing. The major challenges associated with the utilization of biodegradable Mg alloys is their tendency to degrade rapidly at physiological pH and environment containing aggressive ions like Cl^−^ leading to decreased mechanical integrity of the implant before healing^9–11^. Selection of alloying element for biomedical Mg alloys is another major challenge and must be carefully done considering the solubility limit, impurity levels, and biocompatibility^12^. To advance as biodegradable implant materials, appropriate alloying elements selection and processing are crucial. Zinc (Zn) is a primary choice as a secondary alloying element in Mg alloys due to its good solubility (6.2 wt%) in Mg. It can enhance grain refinement, strengthening through solid solution and second phase, while reducing the impact of impurities and increasing passivation film stability. However, Zn additions must be limited to 4 wt% to balance strength and corrosion behaviour^13,14^. Zr, having a solid solubility of 2.69 wt% in Mg, acts as a key refiner of grains in Mg-based alloys incorporating Zn as chief alloying element, improving strength and ductility. It also exhibits good cell adhesion, osteocompatibility, and low ionic cytotoxicity. Usually, the Zr content is restricted to less than 1 wt% in Mg based degradable alloys^15,16^.

Mg alloys have low mechanical properties due to their hexagonal close-packed structure and minimal slip systems, limiting their practical application^18,19^. Severe plastic deformation (SPD) can be effective to improve strength and formability of Mg alloys. SPD produces ultra-fine grains without significant dimensional changes, significantly impacting the mechanical and corrosion properties of biodegradable Mg alloys^20,21^. Multidirectional forging (MDF) is a simpler and less expensive method of SPD that involves continuously upsetting and drawing materials in multiple directions to refine grains and improve mechanical properties. It can be used to create bulk-dense materials on a large scale using existing process equipment^23^. Advanced SPD techniques like equal channel angular pressing (ECAP) and high-pressure torsion (HPT) are proven effective for refining metals and alloys, scaling them up for large-scale industrial use presents significant challenges. Among these SPD techniques, MDF offers ease of use, affordability, and suitability for large-scale industrial goods^24^. Recent studies have highlighted MDF as an effective technique to process Mg alloys by imparting high strain and achieve grain refinement, twinning, dislocation movement, dynamic recrystallization, and texture modification to achieve desirable mechanical properties and corrosion performance^25–27^. Yurchenko et al.^28^ applied a cumulative strain 22.5 through MDF on Mg-0.8Ca, resulting in grain refinement, dynamic recrystallization, and texture modification, leading to increased ultimate tensile strength, ductility, and corrosion resistance. Bahmani et al.^29^ applied MDF to XM11 Mg alloy, revealing increased mechanical strength of close to 7 times high purity Mg. The best corrosion resistance, lesser than high purity Mg was achieved at 300 °C in Mg (OH)_2_ saturated 3.5 wt% NaCl solution.

The implant surface is critical due to its direct interaction with physiological media; thus, significant emphasis must be placed on the development of implant material surfaces. The surface of materials can be altered using a variety of processes, including surface coating, ball burnishing, ultrasonic peening, mechanical attrition for surface treatment and LSP etc^30–34^. One significant benefit of LSP over coating is that it alters the surface of materials without adding new layers or ingredients. The basic principle of LSP involves exposing the metal surface to a short pulse duration laser with a high-power density, causing an absorption layer to absorb laser energy and vaporize, leading to hydrodynamic expansion, high-pressure shock waves, and plastic deformation^35^. LSP can induce compressive residual stress (CRS) and high-density dislocations close to material’s surface, that would enhance mechanical properties. The induced CRS can presumably heal cracks and pores at the surface^36,37^. The combination of CRS and grain refinement caused by LSP can greatly strengthen the material^38,39^. The chloride concentration in an aqueous solution is critical to the corrosion of Mg alloys. Refined grain and compressive residual stress can delay the commencement of corrosion cracking in aggressive environments^40^. Considering its commendable level of industrial success, LSP may be an effective technique for controlling the mechanical and corrosion behaviour of Mg alloys^41^. Zhang et al.^42^ observed LSP improved hardness and strength of AZ31B Mg alloy. Furthermore, LSP did not considerably increase release of Mg^2+^ or weight loss, according to immersion tests conducted in cell culture medium. Additionally, the cell culture study in vitro demonstrated that, LSP processing did not compromise the cytotoxicity of the Mg alloy. Ramanathan et al.^43^ investigated the effect of LSP on Mg-1Zn-0.5Sc alloy and revealed that LSP generated CRS both at the surface and in the subsurface of the Mg-1Zn-0.5Sc alloy, inducing grain refinement via twinning alongside strain hardening and precipitate strengthening. These microstructural changes collectively enhanced the alloy’s mechanical and corrosion properties of the alloy. Additionally, the LSP-treated samples showed improved cell viability and proliferation compared to the as-cast alloy, attributed to uniform apatite layer formation and laser-induced surface texturing that enhanced hydrophilicity and cellular responses. Caralapatti et al.^44^ revealed, corrosion rates of the Mg-0.2% Ca LSP samples were lowered by more than half in contrast with unpeened samples in Hank’ s balanced salt solution and biocompatibility of LSPed samples was also improved.

Recently, few researchers have focused on development of Mg alloys with superior properties through a combination of SPD and post processing methods. As investigated by Kalayeh et al^45^., the combined effect of MDF and post heat treatment of extruded Mg-4Zn-4Al-0.6Ca-0.5Mn resulted in ultrafine grain refinement, and increased ultimate compressive strength and strain. It was also observed that, corrosion rate of the alloy drastically decreased after combined processing. Noteworthy improvement in corrosion resistance of MDF processed, post heat treated Mg-4Zn-1Mn alloy in simulated body fluid (SBF) was observed by Anne et al.^46^, it was ascribed to better spread of secondary phases and refined grain structure. Praveen et al.^47^ employed a combination of Equal Channel Angular Pressing with LSP on AM80 Mg alloy, resulted in refinement of grain in the order of nanoscale leading to increased strength and ductility. LSP also resulted in increased surface hardness and wear resistance.

Recently, a notable increase is witnessed in research aimed at enhancing mechanical and corrosion attributes of Mg alloys, particularly through SPD techniques for bulk grain refinement. In the realm of biomedical applications, it is crucial to not only improve bulk properties but also to enhance surface characteristics. Consequently, employing LSP as a surface modification technique in conjunction with an SPD process like MDF could prove beneficial. Furthermore, there is a scarcity of studies in literature that address the concurrent enhancement of both bulk and surface level properties of Mg alloys. The reported studies have limited their scope to either not exploring the surface properties or have explored only the mechanical strength aspect without considering corrosion aspect. Moreover, the cytocompatibility aspect is also not explored in these studies. This study is different from studies available in the literature as it comprehensively explores bulk as well as surface properties including both strength, corrosion and cytocompatibility study aspects. This multi-faceted performance assessment is critical for biomedical applications of Mg alloys but remains less reported in studies combining severe plastic deformation with laser shock peening, elevating its importance and uniqueness. This paper, therefore, aspires to explore impact of combining MDF and LSP on the mechanical and corrosion performance, as well as the cytocompatibility of Mg-4Zn-0.6Zr alloy specifically designed for biomedical applications.

## Materials and methods

### Alloy preparation and processing

**Fig. 1:**
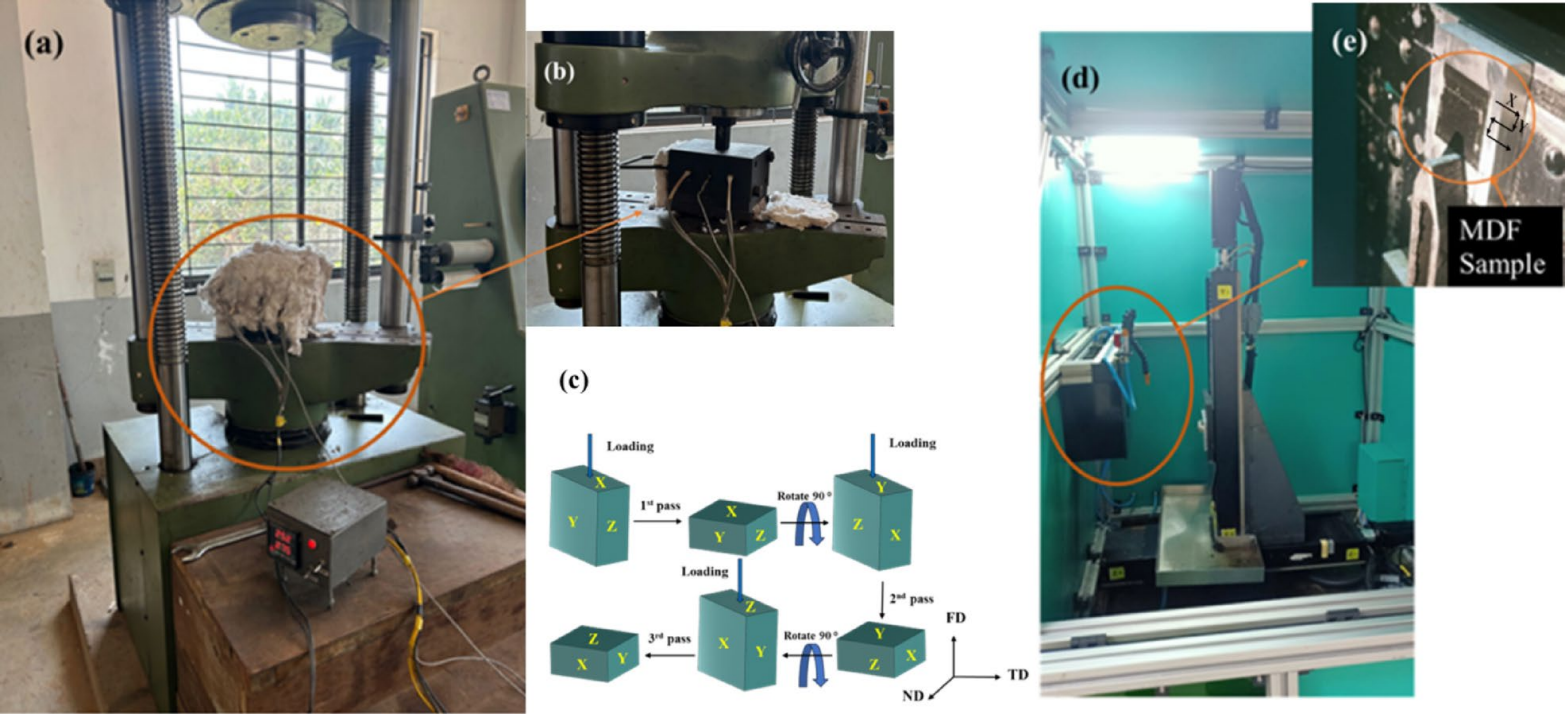
Images (**a**) MDF process containing MDF die and heating element, (**b**) magnified image during loading using UTM, (**c**) schematic representation of MDF process, (**d**) LSP process, (**e**) magnified image showing MDF processed sample mounted for LSP process.

Mg-4Zn-0.6Zr (wt%) ingots were cast in a 125-kW medium frequency induction furnace using 99.9% pure Mg ingots initially and then preheated 99.9% pure Zn ingots and 99.9% pure Zr ingots were are added to the melt. Argon gas environment was maintained in the furnace during the casting. The cast ingots with desired dimensions of 30 mm * 30 mm * 15 mm were machined. The samples were homogenized at 300 °C for 24 h in a tubular furnace. The homogenized samples were made to undergo to MDF at 300 °C using a 600 kN maximum load Universal Testing Machine. A split die was used for MDF process and heating coils were inserted in machined slots of the die. The samples were maintained at 300 °C for about 20 min before each pass. Initially, the load was applied in the X direction and then the sample was removed from the die, rotated about 90° and heated in the die and then the load was applied in the Y direction and then finally load was applied along the Z direction. Five MDF passes were performed with a cumulative strain of 3.45, with a strain of 0.69 during each pass and 0.15 mm/sec pressing speed. A Nd-YAG Q-switched 1064 nm operating frequency, LITRON LSP system was employed to alter the surface properties of MDF alloy. LSP was carried out on the surface 25 mm * 25 mm sectioned from the central portion of MDF processed alloy, along the ND-TD plane which was manually polished using SiC paper upto 1000 grit size. To safeguard the material’s surface from being ablated, an ablation layer consisting of 110 μm black tape was utilized. The parameters selected for LSP: 3 J laser energy, 15 ns pulse width, 3 mm laser beam spot diameter and, 70% overlap. A water overlay of 2 mm thickness was deployed which acted as a plasma-confining medium. The sample nomenclature followed is shown in Table 1 and Fig. 1 represents the MDF and LSP process details.

**Table 1:** Sample code for various processed condition samples.

Sl. No.	Processed condition	Sample code
1	Homogenized Mg-4Zn-0.6Zr alloy	HS
2	1 pass MDF Mg-4Zn-0.6Zr alloy	1-MDF
3	3 pass MDF Mg-4Zn-0.6Zr alloy	3-MDF
4	5 pass MDF Mg-4Zn-0.6Zr alloy	5-MDF
5	5 pass MDF + LSP Mg-4Zn-0.6Zr alloy	5-MDF + LSP

### Microstructural characterization

Microstructural investigations of MDF processed samples were carried out using Leica DM750 Optical Microscope (OM), EVO MA18 with Oxford EDS(X-act) Scanning Electron Microscope (SEM) and [7610FPLUS, Jeol, Japan] Field Emission Gun Scanning Electron Microscope (FEGSEM) with EDS. The samples of 10 mm * 10 mm were sectioned from the centre of the MDF processed samples along the FD-ND plane. The samples were thoroughly polished by SiC papers (400 to 2000 grit size), then by fine cloth polish employing 0.5–1 μm diamond paste, then by acetic-picral solution etching^48^. The average grain size was measured through Image J software. Surface roughness of LSP processed samples were investigated using Innova SPM Atomic Force Microscope. 5-MDF + LSP processed samples were extracted from uppermost superficial layer, the untreated side was ground with grit papers to attain a thickness less than 50 μm. Thinning was ultimately achieved through the use of a dimpling machine (5 μm), succeeded by ion beam milling to generate perforations. JEOL JEM-2100 TEM was used for analysis. Rigaku Miniflex 600 (5th gen) tester was used to carry out X-ray Diffraction (XRD) investigations. Continuous scanning with Theta/2-Theta mode was carried out between 30° to 90° with a step of 0.02°. The samples extracted along the FD-ND plane was utilized for XRD analysis.

### Mechanical properties

Microhardness test was carried out using MATSUZAWA MMTX3 microhardness tester. 10 mm * 10 mm samples used for conducting SEM analysis was used, the samples were initially polished with SiC papers, then by diamond paste employed cloth polish. 100 g load for 15 s dwell period was applied on the samples. Ten readings per sample was noted to obtain average microhardness encompassing both the grain and grain boundaries. Tensile test using Shimadzu universal testing machine (100 kN AG–X plusTM) was conducted. The circular cross section samples for tensile tests were prepared according to ASTM E8M standard (gauge length: 20.0 ± 0.1 mm, diameter: 4.0 ± 0.1 mm, radius of fillet: 4 mm and length of reduced section: 24 mm) and were sectioned out from the centre of the MDF samples along the FD-ND plane. Three samples of each processed condition were tested at 0.5 mm/min speed. The images of fractured samples after the tests were captured using the EVO MA18 SEM. Nano indentation of the LSP processed samples were carried out using KLA Tencor, G200 Agilent nano indenter to estimate the average hardness through five readings at a depth between 600 and 1900 nm from the surface. TB15269 diamond tip indenter was used with a frequency of 45 Hz and a harmonic displacement of 2 nm with strain rate of 0.05 s^−1^. Compressive Residual Stress (CRS) induced by LSP at the surface were measured using PROTO IXRD with MGR40P – X-ray diffraction technique-based stress measurement system with Cr_K-Alpha source, having a wavelength of 2.291 A°. Dual detectors with Beta angles between 20° to −20° were used for measurements.

### Corrosion behaviour and cytocompatibility

The ACM Gill AC electrochemical corrosion setup was used to evaluate the MDF treated samples. Hank’s balanced salt solution 1X (HIMEDIA TL1010) was used for the potentiodynamic polarization and electrochemical impedance spectroscopy measurements, which were conducted at 37 ± 1 °C and pH 7.5. The samples of 10 mm * 10 mm were cut through the centre of all MDF processed samples along the ND-TD plane perpendicular to the last forging direction, the samples were mechanically polished followed by diamond paste assisted cloth polish. 5-MDF + LSP processed sample was subjected to corrosion testing. The saturated calomel served as the reference electrode, a graphite rod as the auxiliary electrode, and a magnesium sample as the working electrode. A electrochemical impedance spectroscopy was conducted with an amplitude of 10 mV in relation to the open circuit voltage and a frequency range of 10,000–0.01 Hz. Cyclic sweep tests were conducted by sweeping through a potential from − 250mV to + 250mV and scan rate of 1mV/s. Tafel extrapolation of the cathodic slope was used to derive the corrosion parameters, such as corrosion potential (Ecorr) and corrosion current density (icorr). Two samples of each condition were tested. FESEM was used to record the corrosion morphology [7610FPLUS, Jeol, Japan].

The cytocompatibility of HS and 5-MDF + LSP samples was evaluated using MG-63 osteoblast-like cells through the MTT assay, employing 3-(4,5-dimethylthiazol-2-yl)−2,5-diphenyltetrazolium bromide. The cells were cultured in Dulbecco’s Modified Eagle’s Medium (DMEM) with 10% Fetal Bovine Serum (FBS) and 1% antibiotic-antimycotic solution. The cultures were kept in a humidified environment at 37 °C and 5% CO_2_ and subcultured using trypsinization once they reached 70% confluence and were utilized for the experiments after going through three successive passages. To assess cytocompatibility, MG-63 cells were seeded at a density of 5000 cells per well in a 96-well plate and incubated at 37 °C in an atmosphere containing 5% CO_2_. The alloy discs were prepared and sterilized by exposure to UV for 20 min before exposure to cells. All procedures were performed under aseptic conditions. After the cells adhered, they were exposed to the prepared alloys for a duration of 24 h. The medium for exposing the alloy discs consisted of Dulbecco’s Modified Eagle Medium (DMEM, Gibco, USA) supplemented with 10% fetal bovine serum (FBS) and 1% penicillin–streptomycin solution. A surface area-to-volume ratio of 1 cm²/mL was maintained for all samples. The pH of the medium was maintained at 7.4 ± 0.2 throughout the procedure. Following the incubation with the test samples, the used medium was removed from the wells, and the cells were treated with MTT reagent (1 mg/mL) and incubated for an additional 4 h at 37 °C. The formazan crystals were dissolved using DMSO, and absorbance was measured at 570 nm with a multimode microplate reader (FluoSTAR Omega, BMG Labtech). The cell proliferation was determined using untreated cells as a reference. Three samples of each condition were used for the study.

Acridine orange-ethidium bromide dual fluorescence staining was employed to distinguish between viable and apoptotic cell populations after treatment with processed samples. To begin, cells were seeded in 24-well plates with 10,000 cells per well density and incubated at 37 °C with 5% CO_2_. Following adherence, the cells were exposed to the prepared alloys for a duration of 24 h. After incubation with the test samples, the cells were stained with 1:1 mixture of acridine orange and ethidium bromide (2 µg/mL each) for 15 min at 37 °C in the dark. Excess stain was ousted with 1X PBS, and the cells were rinsed with 1X PBS. A fluorescence imager (Zoe, Biorad) was utilized to capture images under green and red channels at 20X magnification. Three samples of each condition were used for the study.

## Results and discussion

### Microstructural analysis

#### SEM analysis of homogenized and MDF processed Mg-Zn-Zr alloy

**Fig. 2:**
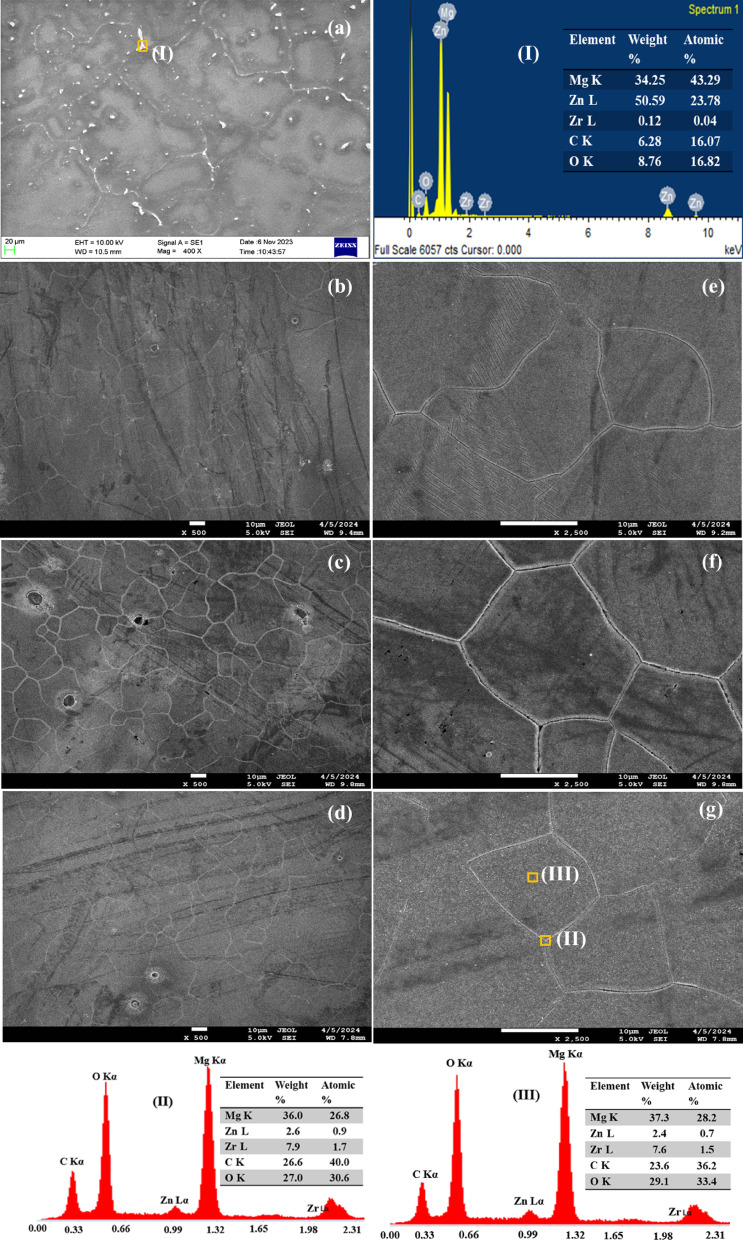
SEM micrographs of (**a**) homogenized sample (HS), (**b**) 1-MDF, (**c**) 3-MDF, and (**d**) 5-MDF processed alloys, magnified views of (**e**) 1-MDF, (**f**) 3-MDF, and (**g**) 5-MDF samples and corresponding EDS spectra (I) HS, (II) 3-MDF, and (III) 5-MDF.

The SEM images of HS and MDF processed samples is presented in Fig. 2. As shown in Fig. 2 (a), the HS microstructure is dendritic in structure and is composed of primary Mg phase and the brightly appearing secondary phases rich in Zn and Zr distributed mostly along the grain boundaries as shown by EDS in zone I. It is also observed that secondary phases are distributed uniformly within the grains throughout the structure. It can be witnessed that HS had grain size of 180 ± 12 μm. A decreasing trend with respect to grain size was witnessed with increased number of MDF passes. After one MDF pass a significant refinement of grains was achieved and grain size was about 27 ± 8 μm as evident in Fig. 2 (b) and 2 (e). It can be noticed that the dendritic structure in homogenized state is fragmented post MDF into equiaxed grains due to induced strain. Figure 2 (c), 2 (f) and Fig. 2 (d), 2 (g) indicates further decrement of grain size after three and five MDF passes to about 20 ± 5 μm and 16 ± 5 μm respectively. A uniform equiaxed distribution of grains was visible throughout the microstructure post higher MDF passes. The refinement of grains after increased number of MDF passes can be ascribed to combined effects of dynamic recrystallization and accumulative strain^49,50^. The EDS spot analysis was carried for 5-MDF sample in the regions II and III, highlighted by (Fig. 2 (g)), one at the grain boundary, other within the grain. It can be seen that the secondary elements Zn and Zr seem to have concentrated more at the grain boundaries as compared to within the grains.

#### TEM analysis of MDF + LSP processed Mg-Zn-Zr alloy

**Fig. 3:**
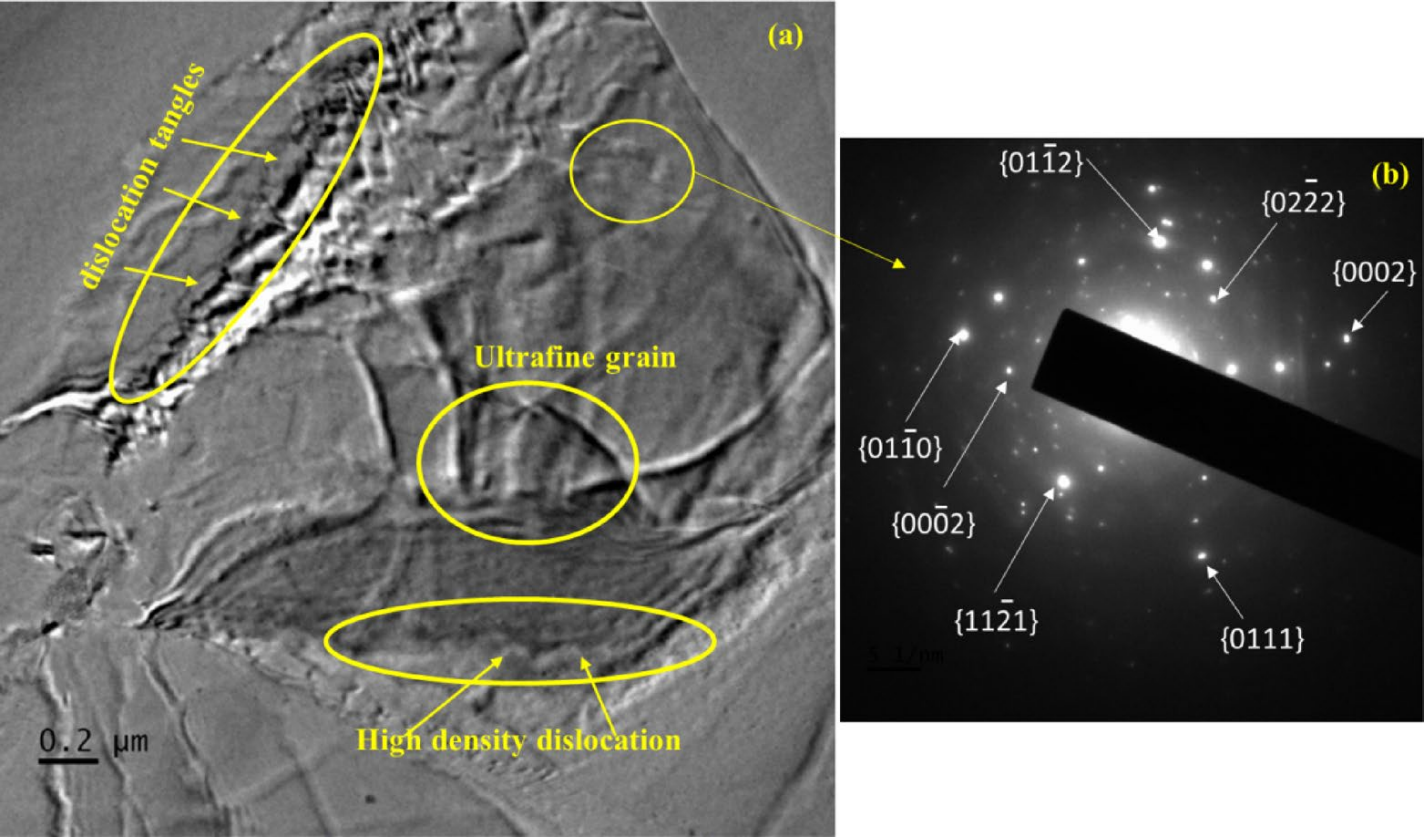
(**a**) TEM micrograph of 5-MDF + LSP processed sample, (**b**) corresponding SAED pattern.

Figure 3. displays the TEM images at the top surface of 5-MDF + LSP processed samples. After LSP treatment, the microstructure of Mg-Zn-Zr alloy at the top surface of the 5-MDF processed sample significantly underwent further more refinement when subjected to laser pulse energy. The corresponding SAED pattern, depicted in Fig. 3(b), a set of continuous diffraction rings appears because LSP introduces a high density of dislocations into the material surface due to SPD^51^. TEM can be employed to observe and analyze dislocation structures, including dislocation walls, tangles, and cells, that emerge from significant plastic deformation as depicted in Fig. 3 (a). Additionally, TEM can detect potential phase transformations that may occur near-surface region due to high-pressure shock waves and their associated thermal effects^52^. High-density dislocation tangles were discovered in the highlighted region, indicating large number of dislocation motions, which were activated due to high strain rate^53^. After SPD, the coarse grains of Mg break down into ultrafine, randomly oriented crystallites. As a result, the diffraction pattern changes from spots to arcs and eventually to continuous rings, confirming grain refinement and orientation randomization^54^. LSP, an effective surface modification technique leads to ultrafine grain layer formation on the surface of Mg alloys which lead to enhanced properties of Mg alloys as justified through nano indentation tests as depicted in Fig. 5 (b) (I).

### XRD phase analysis

**Fig. 4:**
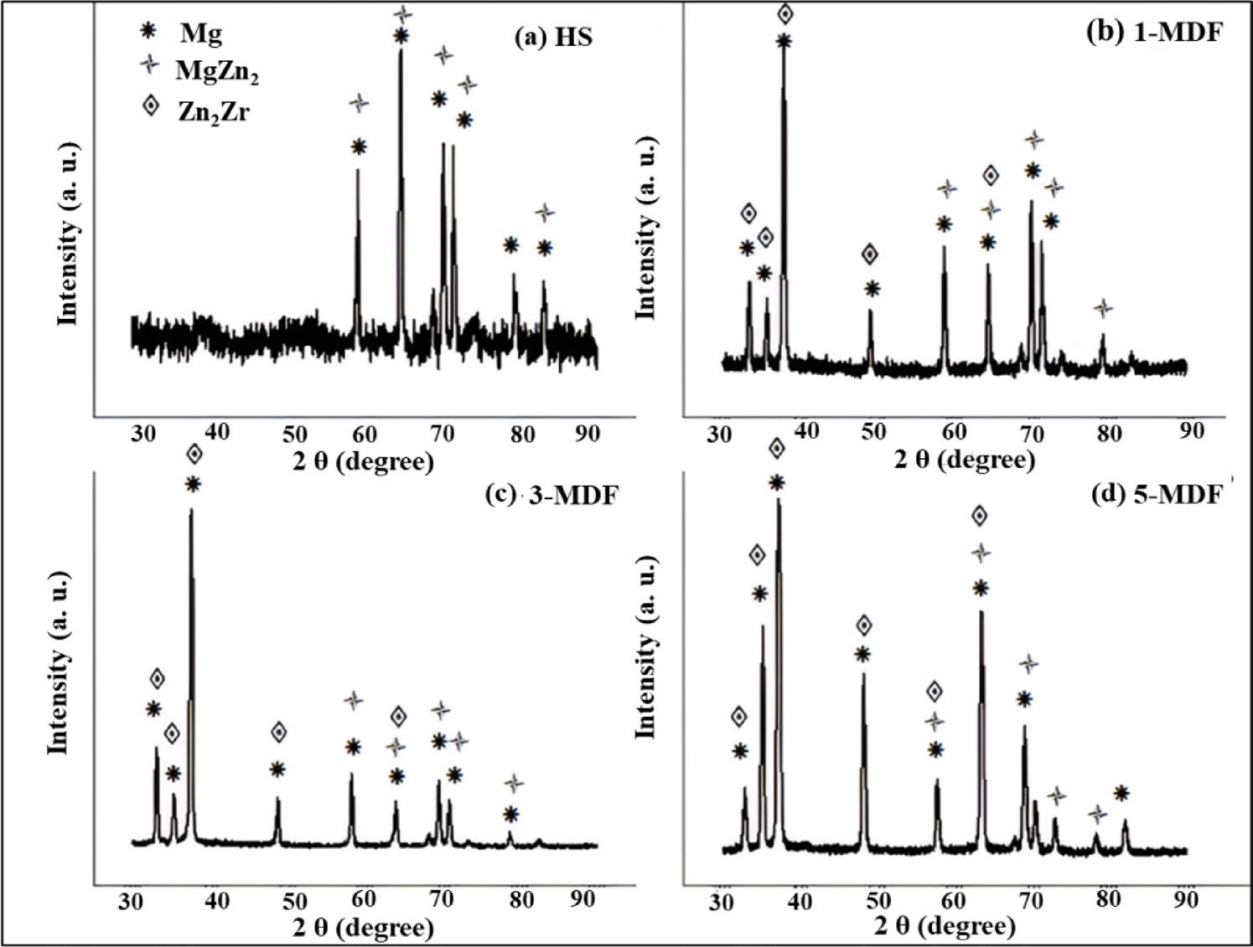
XRD patterns of (**a**) HS, (**b**) 1-MDF, (**c**) 3-MDF and (**d**) 5-MDF processed samples.

XRD patterns used to detect constituent phases contained in the alloy before and after MDF are presented in Fig. 4. It can be understood from the XRD analysis that, the alloy in the homogenized state contained only two phases, primary phase Mg and secondary phase MgZn_2_. However, the MDF processed samples contained one more seconday phase Zn_2_Zr along with previously detected phases. Significant grain refinement induced by MDF redcues crystallite sizes, smaller crystallites can enhance the visibility of certain phases in XRD patterns due to increased phase stability. In the homogenized state, larger grain sizes or a more equilibrium microstructure may suppress the Zn₂Zr phase or make it amorphous, reducing its detectability^55^. The high defect density and localized lattice distortions in MDF samples may stabilize Zn₂Zr, which is not thermodynamically favored in the homogenized state^56^. It can also be observed that the major peak or high intensity peak containing Zn_2_Zr has shifted from 37.82 ° to 37.93 ° and 38.03 ° after one, three and five MDF passes respectively due to higher strain induced by MDF process.

### Mechanical properties

#### Micro hardness, nano hardness and surface roughness

**Fig. 5:**
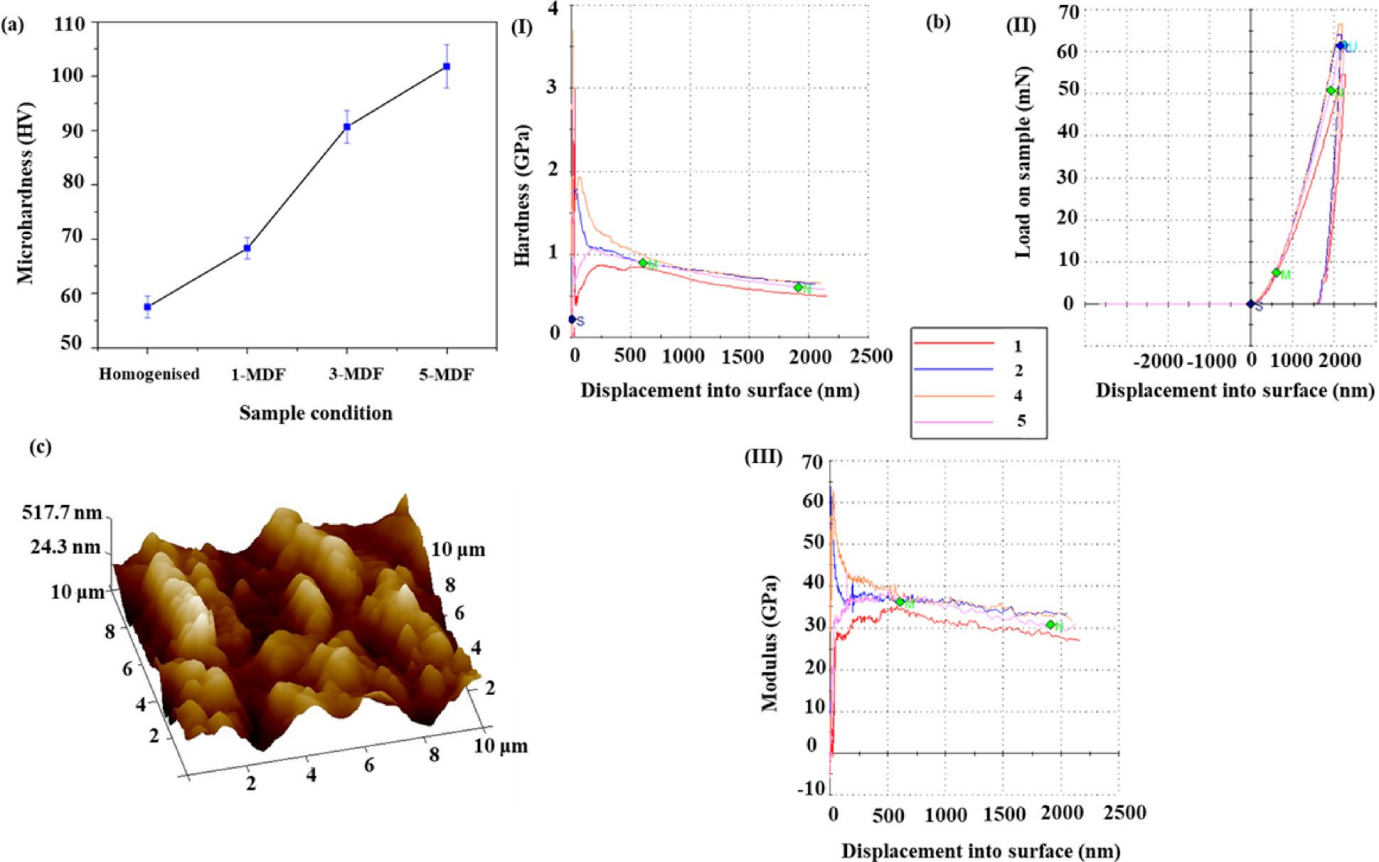
(**a**) Microhardness plot of homogenized sample (HS), 1-MDF, (**c**) 3-MDF, and (**d**) 5-MDF processed alloys, (**b**) nano indentation plots indicating (I) hardness vs. displacement, (II) load vs. displacement and (III) modulus vs. displacement, (**c**) surface topography of 5-MDF + LSP sample.

Figure 5. (a) depicts the variation in the average microhardness values of the homogenized and MDF processed alloys. It is evident that the initial average hardness of HS was 57.7 HV. The average microhardness increased with the increased number of MDF passes from 68.3 HV for 1-MDF to 90.7 HV for 3-MDF and attained a maximum of 101.8 HV for 5-MDF. The initial increment of micro hardness may be due to the combination of strain hardening and grain refinement attained after the first MDF pass. Higher hardness at low stresses is mostly due to increased dislocation densities and their interactions^57^. The increase in microhardness between HS and 1-MDF samples was found to be 18.37%. A significant increase of 32.8% in microhardness was witnessed after three MDF passes than the previous one MDF pass, the highest between consecutive passes. Also, 76.43% increase in microhardness was achieved post five MDF passes from the initial homogenized state. The increment in microhardness may be due to notable grain refinement; the indentation deformation behaviour could be restricted by the grain boundaries^58^. With increased SPD passes, microhardness rises due to grain refining, strain hardening, and homogeneous secondary phases distribution^59,60^. The higher SPD passes, enhance uniform distribution of second phases like MgZn_2_ and Zn_2_Zr. The uniformly distribute precipitates along grain boundaries and within grains during SPD processes, enhances recrystallization which all together improve hardness^61,62^.

The average microhardness of 5-MDF + LSP samples exhibited 112.6 HV, is 95.14% higher compared to HS. Moreover, hardness of 5-MDF + LSP sample improved by further 10.6% as compared to 5-MDF samples mainly due to increased density of dislocations and refined grains observed near the peened surface after treatment of LSP, which is observed in TEM analysis (Fig. 3) and induced residual stress during LSP. LSP-induced sub-grain development and twin boundaries contribute to higher surface hardness. As it gets farther away from the surface, hardness decreases, this is because of LSP produces plastic strain with a gradient character. Gradient work-hardening and microstructure evolution result from the plastic strain being highest at the top surface and progressively decreasing deeper into the interior^47^.

Figure 5 (b) presents the 5-MDF + LSP samples data obtained from the nano indenter, where experiments were performed to a depth of around 2000 nm at 0.05 s^−1^ strain rate, using tip TB15269. Figure 5 (b I) depicts the relationship between load and displacement of a nano indenter as it penetrates a material capped at 2000 nm. The studies used a high-precision load cell and a capacitor-based transducer to measure displacement. The indenter penetrated 45 times per second, with a 2 nm step size. Figure 5 (II & III) illustrates the depth-wise profile of hardness and modulus, with each experiment conducted five times to mitigate surface variability. The 5-MDF + LSP samples exhibited an average hardness of 0.76 GPa and modulus of 34.2 GPa. As the depth increases, both hardness and modulus values decrease, indicating the influence of LSP on the Mg-Zn-Zr alloy.

The surface morphology of MDF 5P + LSP sample is shown in Fig. 5 (c) and values of *Ra* and *Rz* obtained from the AFM topography were 119 nm and 152 nm respectively. LSP significantly alters the topography of Mg alloys by creating dents, refining grains, increasing hardness, inducing compressive residual stress, and improving corrosion resistance. These changes are crucial for elevating mechanical and surface properties of Mg alloys^63^.

#### Tensile performance and fracture analysis

**Fig. 6:**
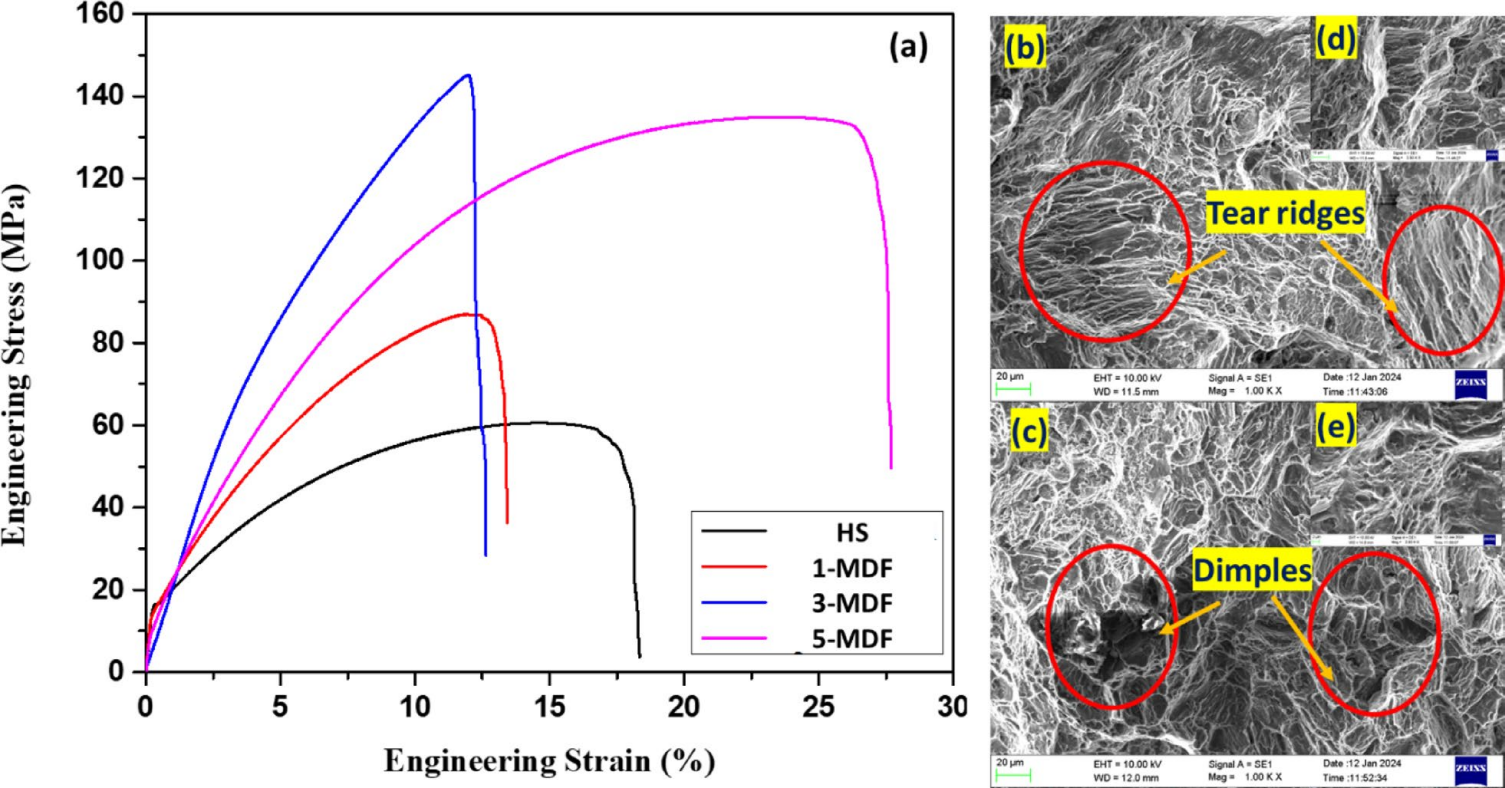
(**a**) Engineering stress vs. strain curves of HS and MDF processed samples, fractography of (**b**) HS and (**c**) 5-MDF samples, magnified fractography views of (d) HS and (e) 5-MDF samples.

Figure 6 presents the stress-strain plot of the homogenized and MDF processed alloy specimen. HS exbhited least mechanical strength with UTS of 60 MPa, while the UTS of 1-MDF was found to be 87 MPa, a 45% increase. The maximum UTS of 145 MPa achieved was for 3-MDF which was 141% increase in comparision to HS. 5-MDF was able to attain UTS of 134.9 MPa which was an increment of about 125% as compared to HS. The elongation of HS was 14%, 1-MDF and 3-MDF samples could attain elongation values slightly lesser compared to HS, 11.88% and 11.99% respectively. However a significant increase in elongation of 23.48% was obatined for 5-MDF samples, surpassing all previous conditon alloys. The peak UTS achieved at 3-MDF can be attributed to grain refinement and strain hardening, while the highest elongation at 5-MDF arises from synergistic outcome of greater recrystallization, larger strain accumulation and texture modification that enhance ductility with some trade-off in tensile strength^64,65^.

Fractured morphologies of samples after homogenization and five pass MDF samples post tensile test is depicted in Fig. 6 (b-e). Huge cleavage planes, tear ridges, very few dimples along with casting defects would lead to a typical brittle mode of fracture as observed in Fig. 6 (b and d) representing HS fractured surface. The cleavage fracture primarily results because of limited slip systems of Mg alloys^66,67^. Figure 6 (c and e) representing the 5-MDF fractured surface clearly indicates presence of large number of dimples an indication of failure due to superior elongation. Fracture elongation by crack propagation inhibition can be improved by finer grains, which could be the reason why fine-grained 5-MDF samples exhibited highest ductility^68^. Increasing the number of MDF passes caused progression in tensile properties for the considered Mg-Zn-Zr alloy. However, this improvement was more remarkable in higher MDF pass samples, due to the substantial grain refinement by Zr-rich particles^69^. The combination of refined grains and precipitate strengthening from MgZn_2_ and Zn_2_Zr phases forms a composite strengthening mechanism. The MgZn_2_ precipitates act as barriers to dislocation motion, resulting in enhanced hardness, yield strength, and tensile strength in the alloys^61^. The presence of Zn_2_Zr precipitates along with MgZn_2_ phases contributes to improved strength through dispersion and precipitation hardening in SPD-processed alloys^70^. The 5-MDF sample’s fracture surface (Fig. 6c) exhibited fine, deep dimples, revealing a full ductile fracture mode with an EL of 23.48%.

#### Compressive residual stress

**Fig. 7:**
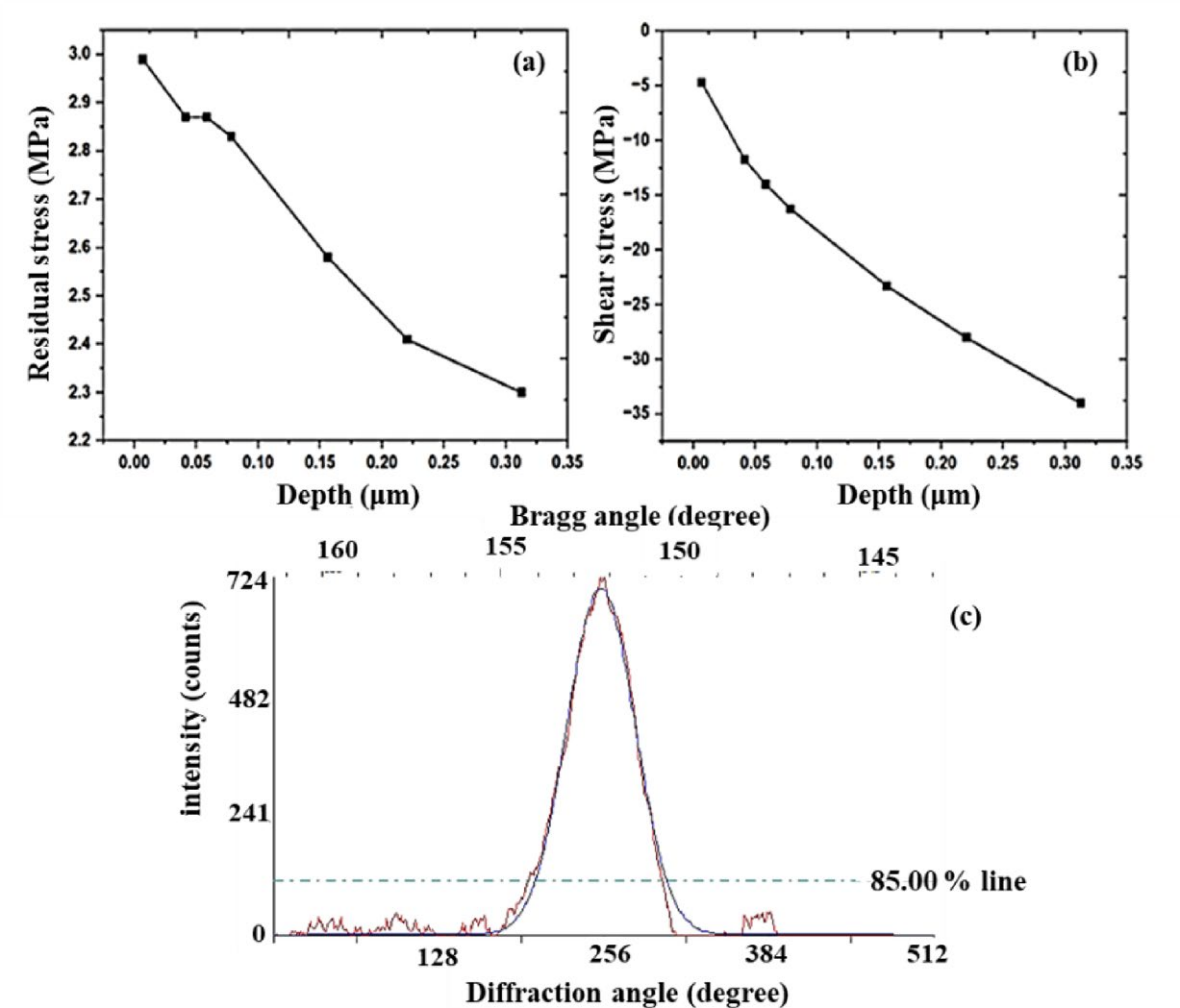
(**a**) Residual stress distribution, (**b**) shear stress of the 5-MDF + LSP specimen with distance and (**c**) diffraction peak plot.

Figure 7. (a) displays residual stress distribution in the MDF 5P + LSP samples. A high-level introduction of residual compressive stress at the surface of LSP treated surface, achieving a maximum of about − 2.87 MPa was observed. The MDF 5P + LSP sample had an average residual stress value of 2.6 MPa; as the depth increases, residual stress values decrease, as shown in Fig. 7 (a). The results were in mutual agreement with nanoindentation profile (Fig. 5 (b)) and the residual stress profiles vs. the depth almost follows the similar trend. In this investigation, residual stress profiles for MDF 5P + LSP specimens were generated as function of depth, as shown in Fig. 7 (a). According to Rai et al.^71^, following LSP, some evidence of thermal flaws and a greater amplitude of compressive residual stress were discovered right at the surface. Figure 7 (b) shows the profile of shear stress; the shear stress profile is as intriguing as the residual stress profile. The shear stress falls as the effective depths rise. The diffraction peak plot indicated by Fig. 7 (c), the diffraction peak shift that is used to determine the residual stress on the material surface.

According to Rossini et al.^72^, residual stress can be controlled by reducing or eliminating it, or by purposefully inducing advantageous CRS at the surface and subsurface levels using methods like LSP. SPD is involved in the LSP-induced beneficial CRS, which improves wear and corrosion resistances and prevents fatigue cracks from origination and propagation. LSP-induced residual stresses fall into the macro and micro stress categories. CRS can enhance the biocompatibility of implant materials. For example, shot peening and other mechanical surface treatments that introduce CRS can improve surface properties of Mg alloys, making them more conducive to cell adhesion and growth. This is crucial for biomedical applications wherein interaction between surface of the implant and biological tissues is critical^73^.

### Electrochemical corrosion analysis

**Table 2:** Details of corrosion parameters.

Sample condition	Rest potential (mV)	I_corr_ (ma/cm^2^)	B_a_ (mV)	B_c_ (mv)	*R*_sol_	*R*_ct_ (omh cm^2^)	Corr rate (mm/yr)
H	−1453.8	0.3910	41.23	787.9	1.65E + 02	5.72E + 02	0.0540
1-MDF	−1513	0.2020	27.351	176.28	1.93E + 01	1.45E + 03	0.0279
3-MDF	−1496.2	0.1576	16.064	81.683	1.63E + 01	4.03E + 03	0.0217
5-MDF	−1461.6	0.1683	13.412	91.954	1.74E + 01	7.94E + 03	0.0232
5-MDF + LSP	−1405.1	0.0346	24.591	166.21	1.47E + 01	8.83E + 03	0.0047

**Fig. 8:**
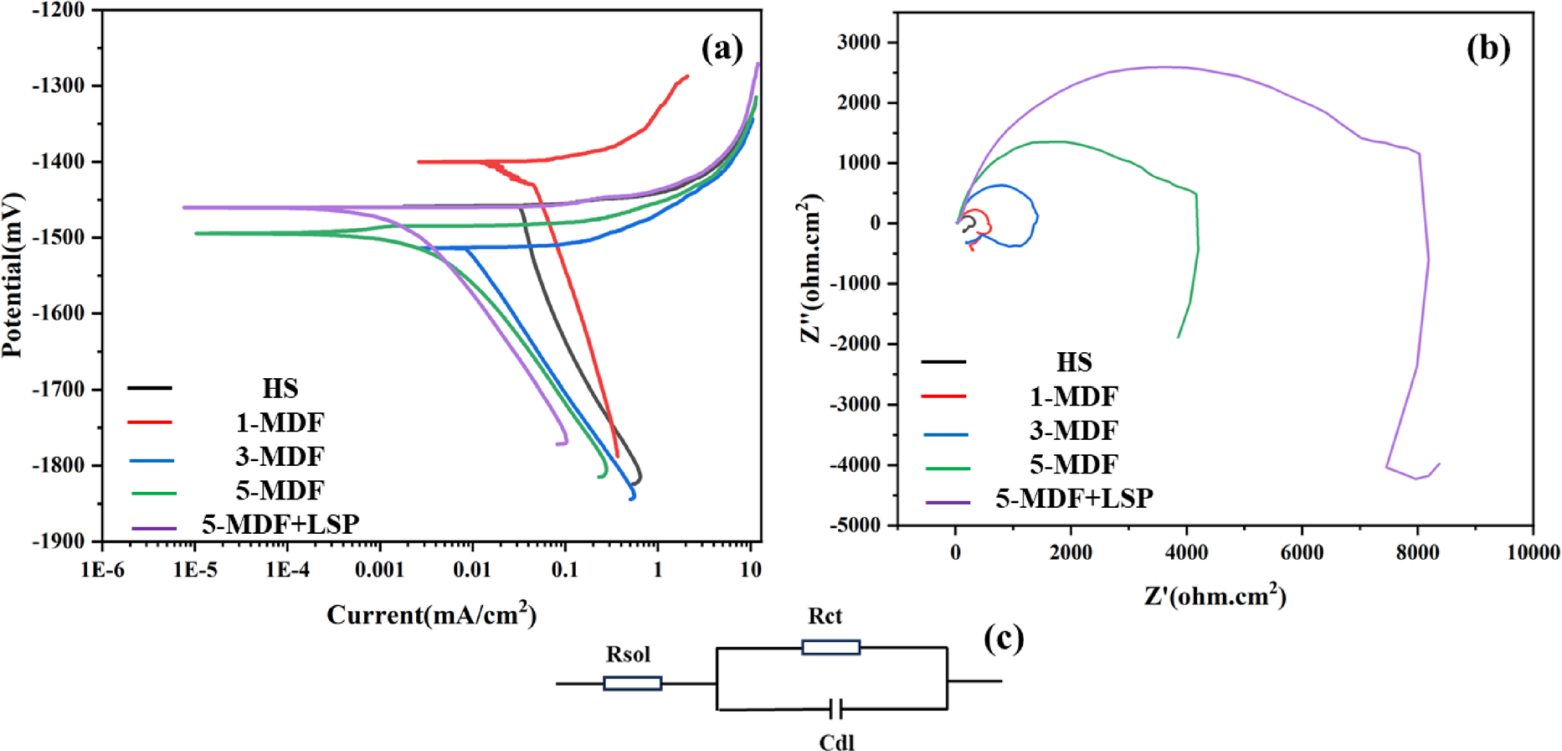
(**a**) Tafel plots of HS, MDF processed and MDF + LSP processed samples, (**b**) corresponding Nyquist plots and (**c**) equivalent circuit.

Figure 8 (a) displays the potentiodynamic polarization plots of all samples including HS, 1-MDF, 3-MDF, 5-MDF and 5-MDF + LSP. According to the results, it was observed that, corrosion current density diminished with increased MDF passes as compared to HS and the least corrosion current density was obtained for 5-MDF + LSP samples. The 5-MDF + LSP processed samples exhibited a corrosion potential of – 1405.1 mV, the noblest among all the considered samples and a least corrosion current density of 0.0346 mA/cm^2^. Also, the corrosion rate for HS was found to be 0.0540 mm/yr which decreased after MDF processing and least corrosion rate of 0.0047 mm/yr was attained for 5-MDF + LSP sample. From, the obtained results, it can be derived that the samples processed with a combination of both MDF and LSP process can substantially improve corrosion resistance of Mg alloys; in this case, it was found to be about a factor of one order of magnitude reduction in corrosion rate. Figure 8 (b) provides an insight to the Nyquist plots of the considered samples. Through the plots it can be inferred that larger diameter of capacitance loop indicates superior corrosion resistance offered by respective processed samples^74,75^. As evident, the capacitance loop of HS is smallest and diameter of capacitance loop of the MDF processed alloys increase with number of passes. The highest diameter capacitance loop is obtained for the 5-MDF + LSP sample, which signifies best corrosion resistance and agrees with results obtained through potentiodynamic polarization tests. The equivalent circuit of the electrochemical impedance spectroscopy is shown in Fig. 8 (c) where in R_sol_ in series with a parallel combination of R_ct_ and C_dl_. R_sol_ represents the ohmic resistance through which ions move in the solution, R_ct_ represents the resistance to the electrochemical reactions such as oxidation or reduction on the electrode surface and C_dl_ represents the capacitance of the electric double layer formed at the electrode-electrolyte interface. As observed in from Table 2, the HS has the least R_ct_ and highest R_sol_ exhibiting the least corrosion resistance among the considered samples. The increased MDF passes resulted in increased R_ct_ values indicating improved resistance to charge transfer thus exhibiting better corrosion performance and 5-MDF + LSP processed samples exhibited the best corrosion inhibition among the considered alloys.

**Fig. 9:**
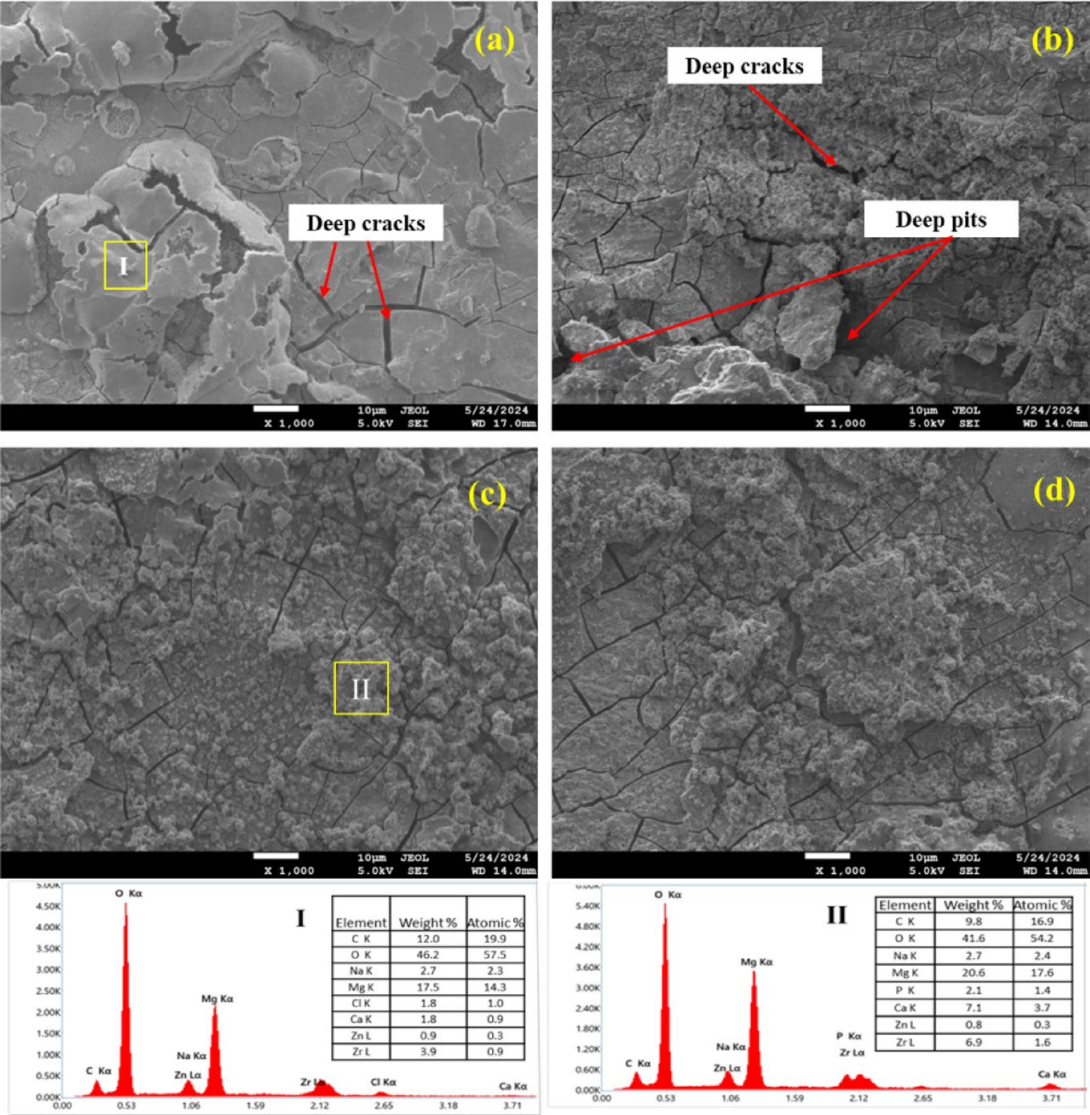
SEM images of corrosion test (**a**) HS, (**b**) 1-MDF, (**c**) 3-MDF, (**d**) 5-MDF, (I) and (II) are EDS of HS and 3-MDF respectively.

As depicted by Fig. 9 (a), the corrosion morphology of HS can be seen with deep cracks at several locations which would allow the penetration of SBF and result in non-uniform and rapid degradation. Figure 9 (b) presents the corrosion morphology of 1-MDF where in along with deep cracks, deep pits can be observed at couple of sites again leading to poor corrosion resistance compared to MDF processed samples. Figure 9 (c) and (d) depicts corrosion morphology of 3-MDF and 5-MDF samples, it was witnessed that uniform fine grain structure and distribution of products of corrosion on the surface of these samples within no deep cracks or pits leads to reduced corrosion rate. The products of corrosion themselves form a protective layer resisting SBF penetration into the surface of the samples thereby delaying the corrosion. Calcium and Phosphorous detected in the corrosion products especially in 3-MDF sample also indicates better bioactivity^76^, similar observation can be made in 5-MDF sample. The corrosion morphology for MDF alloy post processed through LSP is not available but the further improvement in corrosion rate as depicted in Table 2 could be a result of nano grained structure^40^, induced compressive stresses that can heal the cracks and pores^36,37^.

### Cytocompatibility behavior of MDF + LSP processed Mg-Zn-Zr alloy

Results of cytocompatibility testing performed using MG-63 osteoblast-like cells demonstrated the cell proliferative efficacy of the 5-MDF + LSP samples. Compared to HS alloys, 5-MDF + LSP processed alloys showed enhanced cell proliferative potential implying their potential role in bone cell proliferation and regeneration (Table-3). Numerous modified alloys have been shown to enhance the growth of osteoblasts. Surface alterations that can boost the material’s osteoinductivity and biocompatibility are frequently used to accomplish this strategy. Osteoblast adhesion, proliferation, and differentiation are stimulated by several specific techniques and alloy combinations, which eventually aid in bone tissue repair and regeneration^77^. Bone regeneration is known to be stimulated by Zn, a necessary trace metal. It is essential for collagen production, osteoblast differentiation, and alkaline phosphatase activity, all of which support efficient bone repair. Based on results of this study, the Zn-alloyed Mg alloys increased osteoblast attachment and proliferation during the 24 h of incubation, which is in line with earlier research showing that Zn has a beneficial effect on osteogenic activity^78^. Alternatively, Zr is shown to boost mechanical characteristics and corrosion resistance of Mg alloys, despite not being a naturally occurring trace element in bone. Zr presence seemed to slow down the alloy’s rate of degradation in as evident through this investigation, creating a more stable platform for cell development. Rapid pH increase and hydrogen evolution are common outcomes of excessive Mg corrosion, both of which are detrimental to cell survival. Therefore, the incorporation of Zr seems to be essential for balancing biocompatibility and degradation kinetics, which in turn fosters favourable conditions for osteoblast development^79^.

**Table 3:** Cell proliferative potential of the fabricated alloys on MG-63 osteoblast-like cells.

Material	Cell proliferation % (Mean ± SD)
Control	100 ± 0.00
HS	97.55 ± 1.52
5-MDF + LSP	113.86 ± 1.31

#### Live-dead assay using dual fluorochrome staining

**Fig. 10:**
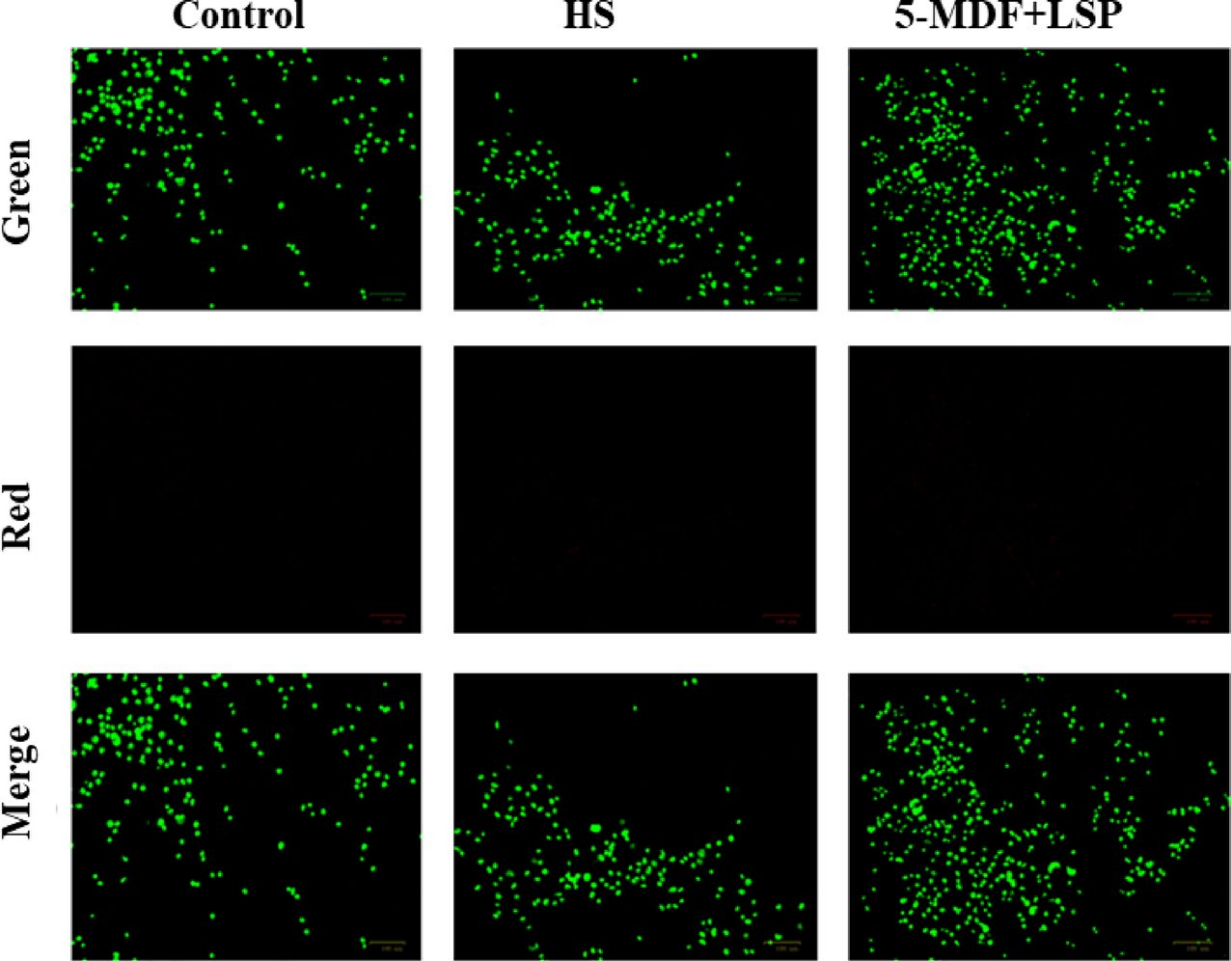
Photomicrographs of MG-63 osteoblast-like cells exposed to control cells, HS and 5-MDF + LSP. Green channel indicates nuclei stained with acridine orange and red channel indicates nuclei stained with ethidium bromide (Magnification 20X).

Acridine orange stains the viable nuclei fluorescing green, while ethidium bromide staining indicates the apoptotic nuclei, fluorescing red. Hence, under the merge channel, green fluorescence indicates the presence of live cells whereas yellow-green nuclei indicate the presence of early-apoptotic cells. Results of this study indicated that the 5-MDF + LSP alloys promoted cell viability and proliferation, confirmed by the presence of green nuclei in the merge channel and the absence of ethidium bromide-stained nuclei as indicated by Fig. 10.Although dual staining offers qualitative information about the distribution of viable and dead cells, the outcomes closely align with the quantitative evaluations from complementary tests like MTT. Further, the results demonstrate exceptional biocompatibility in promoting osteoblast growth and proliferation of Mg-Zn-Zr alloy processed with combination of MDF and LSP, which may make them promising avenues for biodegradable orthopaedic implant applications.

Through LSP processing of the MDF processed alloy, better surface properties could be achieved at the surface and sub-surface of the considered Mg-Zn-Zr alloy. The surface roughness of the LSP treated alloy is an important contributor to achieve desired cell adhesion onto the substrate surface. The LSP tailored surface exhibited the best corrosion properties amongst the samples considered which may be attributed to grain refinement and beneficial compressive residual stresses imparted by LSP^80^. Slower degradation rate leads to controlled release of substrate ions such as Mg^2+^ and Zn^2+^ which in turn are biocompatible thereby facilitating the growth and proliferation of MG-63 osteoblast-like cells^80^.

## Conclusion

The combination of MDF as a bulk deformation severe plastic deformation process and LSP as a surface modification technique applied to develop Mg-4Zn-0.6Zr alloy for biodegradable implant application resulted in several key conclusions highlighted as follows:

1. MDF achieved substantial grain refinement and uniform equiaxed grain distribution with increased number of passes, initial average grain size of HS was 180 ± 12 μm, and after 5 MDF passes the average grain size was 16 ± 5 μm, which may be ascribed to combined effects of accumulative strain and dynamic recrystallization. The 5-MDF alloy subjected to LSP resulted in further refinement of grains to nano scale with high density of dislocations and dislocation tangles as depicted by TEM.
2. MDF processed samples resulted in significant increase of mechanical properties, average microhardness of 101.8 HV was achieved for 5-MDF sample which was 57.7 HV for HS, an increase of 76.43% and 5-MDF + LSP samples achieved an increase of about 95% with average microhardness of 112.6 HV. Similarly, the 5-MDF sample achieved a tensile strength improvement of about 125% compared to HS and maximum elongation of 23.48% was attained for 5-MDF.
3. The nano indentation tests and compressive residual stresses for 5-MDF + LSP samples indicated that the surface properties were greatly enhanced by LSP treatment post MDF.
4. The electrochemical corrosion tests in HBSS indicated that the MDF processed samples at higher passes achieved significant reduction in corrosion rate with reduced corrosion cracks and pits, uniform distribution of corrosion products forming a resistive layer for penetration of SBF, thereby delaying corrosion. The 5-MDF + LSP sample displayed the best corrosion resistance with about a factor of one order of magnitude reduction in corrosion rate, mainly attributed to LSP induced nano refinement of grains and compressive residual stress.
5. Cytocompatibility tests with MG-63 osteoblast-like cells revealed that the 5-MDF + LSP samples were cell proliferative. MDF + LSP treated alloys outperformed bare alloys in terms of cell proliferative potential, revealing a possible involvement in bone cell proliferation and regeneration. The alloying elements Zn and Zr have shown improved adhesion and proliferation of cells in a live-dead assay using dual fluorochrome staining.

The above results indicate that the MDF + LSP processed Mg-4Zn-0.6Zr alloy could be a potential candidate for temporary biodegradable implant application, further in vivo testing of the developed alloy may be explored in the future.

## Data Availability

The datasets generated and/or analysed during the current study are not publicly available but are available from the corresponding author on reasonable request.
